# Editorial Note: An analysis of trade cooperation: Central region in China and ASEAN

**DOI:** 10.1371/journal.pone.0335696

**Published:** 2025-10-30

**Authors:** The PLOS One Editors

This article [1] was identified as one of a series of submissions for which we have concerns that the peer review did not meet the expected standards for *PLOS One*. We regret that the issues were not identified and addressed prior to the article’s publication.

This note is not intended to question the quality of the article or contributions of the authors to the review process.
